# Binding Study of the Fluorescent Carbazole Derivative with Human Telomeric G-Quadruplexes

**DOI:** 10.3390/molecules23123154

**Published:** 2018-11-30

**Authors:** Agata Głuszyńska, Bernard Juskowiak, Błażej Rubiś

**Affiliations:** 1 Laboratory of Bioanalytical Chemistry, Faculty of Chemistry, Adam Mickiewicz University, Umultowska 89b, 61-614 Poznań, Poland; juskowia@amu.edu.pl; 2 Department of Clinical Chemistry and Molecular Diagnostics, University of Medical Sciences, Przybyszewskiego 49, 60-355 Poznań, Poland; blazejr@ump.edu.pl

**Keywords:** G-quadruplex, telomere, carbazole derivative, biological activity, spectroscopy, telomerase

## Abstract

The carbazole ligand **3** was synthesized, characterized and its binding interactions with human telomeric (22HT) G-quadruplex DNA in Na^+^ and K^+^-containing buffer were investigated by ultraviolet-visible (UV-Vis) spectrophotometry, fluorescence, circular dichroism (CD) spectroscopy, and DNA melting. The results showed that the studied carbazole ligand interacted and stabilized the intramolecular G-quadruplexes formed by the telomeric sequence in the presence of sodium and potassium ions. In the UV-Vis titration experiments a two-step complex formation between ligand and G-quadruplex was observed. Very low fluorescence intensity of the carbazole derivative in Tris HCl buffer in the presence of the NaCl or KCl increased significantly after addition of the 22HT G4 DNA. Binding stoichiometry of the ligand/G-quadruplex was investigated with absorbance-based Job plots. Carbazole ligand binds 22HT with about 2:1 stoichiometry in the presence of sodium and potassium ions. The binding mode appeared to be end-stacking with comparable binding constants of ~10^5^ M^−1^ as determined from UV-Vis and fluorescence titrations data. The carbazole ligand is able to induce formation of G4 structure of 22HT in the absence of salt, which was proved by CD spectroscopy and melting studies. The derivative of carbazole **3** shows significantly higher cytotoxicity against breast cancer cells then for non-tumorigenic breast epithelial cells. The cytotoxic activity of ligand seems to be not associated with telomerase inhibition.

## 1. Introduction

It is known that human genomic DNA contains guanine rich sequences capable of building stable four-stranded structures called G-quadruplexes (G4s) [1]. They are made of two or more stacks of four co-planar guanines connected by Hoogsteen-type hydrogen bonds. G4s are stabilized by stacking interactions between G-tetrads, physiological concentration of monovalent cations (sodium or potassium), and/or by small ligands with characteristic structural features [2,3,4]. The interest in G-quadruplexes has increased after the publication of reports on the existence of G-quadruplex structures in human cells [5,6,7], and their supposed regulatory roles in biology [8]. The structures of G-quadruplex are important in promoter regions of oncogenes such as c-MYC [9,10], c-KIT [11,12], bcl-2 [13,14], RET [15,16], or VEGF [17], as well as in human telomeres [18]. The telomeres end with a single-stranded DNA of the repeatable sequence (TTAGGG)_n_ that exists at both ends of eukaryotic chromosomes in human cells. In a normal cell telomeres get shortened upon each cell division, so they act as a special biological clock and restrict the number of possible replications [19,20,21]. In up 85% of cancer cells, telomere shortening is compensated by telomerase, a ribonucleoprotein (RNP) complex reverse transcriptase, so the tumor cells are effectively immortalized [22]. Since the first reports on G-quadruplex ligand activity (inhibition of human telomerase) [23,24], the number and variety of “G-quadruplex ligands” has increased rapidly over the years [2,3,25,26,27,28,29,30,31,32,33,34,35,36,37,38,39,40,41,42,43,44,45]. Among them are derivatives of carbazole, for example 3,6-bis[2-(1-methylpyridinium)vinyl]carbazole (BMVC) [33,34,35,36], benzimidazole‒carbazole ligands [37,38,39] and others [40,41,42,43,44,45]. Carbazole derivatives have aroused much interest because of their photophysical [46,47,48,49,50,51] and biological properties [52,53,54]. Imidazole group and carbazole derivatives have been shown to exhibit various biological properties and therefore play important role in medicinal chemistry [55,56,57,58,59].

Recently, we have reported on the stability and interaction of three carbazole ligands with G-quadruplexes formed by sequences corresponding to region of c-MYC NHE III_1_ and c-KIT 1 [43,44]. It has been found that introduction of different substituents on the nitrogen atom of carbazole has modest impact on the G-quadruplex binding properties as the relevant ligand demonstrated similar stabilization of G4s and comparable binding affinities in the order of 10^5^ M^−1^. In this study we use ultraviolet-visible (UV-Vis) spectrophotometry, fluorescence and circular dichroism (CD) spectroscopy to study the interaction of our most promising carbazole ligand carrying imidazole moiety with G-quadruplex formed by a telomeric sequence in the presence of sodium and potassium ions_._ Under physiological conditions the human telomeric sequence d[AGGG (TTAGGG)_3_] (22HT) has been found to form different types of G-quadruplex structures. In Na^+^ solution an intramolecular antiparallel basket-type G-quadruplex structure with one diagonal and two lateral loops was observed by nuclear magnetic resonance (NMR) spectroscopy [60]. In K^+^ environment depending on the method used, completely different structures of the same sequence were obtained. The crystallographic studies in the presence of K^+^ showed a parallel structure [18], while the use of NMR spectroscopy in K^+^ solution showed existence of two distinct (3 + 1) topologies, termed hybrid-1 and hybrid-2 G-quadruplex structures [61,62,63,64]. Furthermore, we report the ligand-induced inhibition of telomerase activity assessed by a telomerase repeat amplification protocol assay (TRAP), as well as cytotoxic activity against breast cancer and non-cancer cells.

## 2. Results and Discussion

### 2.1. Chemistry

**Figure 1:**
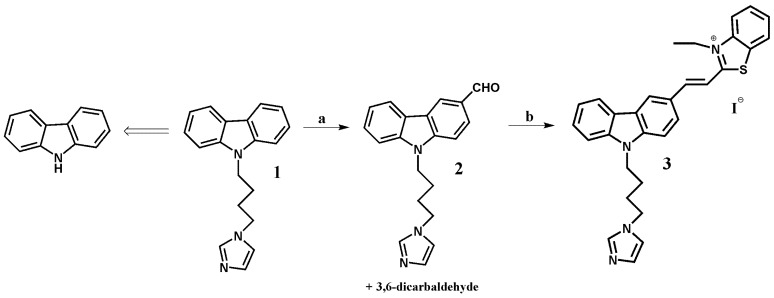
Reagents and conditions for the ligand **3** synthesis: (a) POCl_3_, DMF, Cl(CH_2_)2Cl, 0 °C → 25 °C (2 h), 90 °C (32 h); (b) 3-ethyl-2-methyl-benzothiazolium iodide, MeOH, 25 °C, 4 h.

In this paper we report the synthesis of a (*E*)-2-(2-(9-(4-(1*H*-imidazol-1-yl)butyl)-9*H*-carbazol-3-yl)vinyl)-3-ethylbenzo[*d*]thiazol-3-ium ligand **3**, which is capable of stabilizing G-quadruplex structures (Figure 1) [43,44]. The starting 9-(4-(1*H*-imidazol-1-yl)butyl)-9*H*-carbazole **1** was synthesized from carbazole by a known synthetic procedure in 62% overall yield [65].

The Vilsmeier–Haack reaction is one of the methods for the preparation of aldehydes by creating new carbon-carbon bonds. In order to synthesize compound **2**, we have modified the known procedures for the formylation of carbazole compounds, using a large excess of reagents and conducting the reaction for 32 h. [66,67,68,69,70]. Shorter reaction time had an impact in terms of poor efficiency, while a significant prolongation of reaction time resulted in the formation of only bisaldehyde with poor yield and other by-products. Ligand **3** was prepared by Knoevenagel type condensation between monoaldehyde **2** and 3-ethyl-2-methylbenzothiazolium iodide in methanol. Reaction was carried out without piperidine as a basic catalyst (pKa 11.22) [71]. The basic nature of imidazole (pKa 14.5), was sufficient to the progress of the reaction. Experiments with the addition of piperidine proceeded with a lower yield. Earlier synthesis reactions of carbazole derivative with the triazole (pKa 10.3) substituent required the use of piperidine [45]. The structure of the final product was established in the course of the ^1^H-NMR spectrum. Its *E* configuration was determined on the basis of a value of the CH=CH coupling constant (*J* = 15.4 Hz) doublets at 8.02 and 8.42 ppm. The confirmation of structure was the electrospray mass spectrum (ES-MS) and the presence of signal *m*/*z* 477 [M]^+^ in a positive mode and *m*/*z* 127 [M]^−^ in a negative mode. The high-performance liquid chromatography (HPLC)-pure ligand was used without further purification.

### 2.2. Spectral Properties of Ligand 3

**Figure 2:**
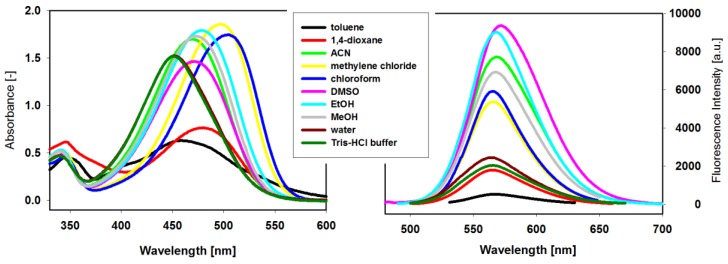
Absorption ([3] = 3.6 × 10^−5^ M) and fluorescence ([3] = 2.2 × 10^−6^ M) spectra of the dye **3** in selected organic solvents and aqueous solutions at room temperature (λ_ex_ = Abs. λ_max_).

In order to investigate the effect of different solvents on the UV–Vis and fluorescence spectra properties, the stock solutions of compound **3** in dimethyl sulfoxide (DMSO) were diluted by different solvents. The new ligand **3** is soluble and stable in selected organic solvents and aqueous solutions (Figure 2). The UV-Vis absorption spectra have long-wavelength absorption band characteristic for the coupling of carbazole with benzothiazolium group by a double bond carbon-carbon. Large solvent-dependent shifts (53 nm) and variations in molar absorptivities were observed, and they seemed to correlate with the polarity of the aprotic and protic solvents (Table 1). The wavelengths of absorption band in CH_2_Cl_2_ and CHCl_3_ were above 500 nm (501 and 504 nm, respectively), while the wavelengths in other solvents were below this value and the wavelengths in H_2_O and in TrisHCl buffer were the lowest. The absorption band of the ligand solution in nonpolar toluene possesses much lower intensity and clearly is blue-shifted (452nm), which suggests the formation of dimers or higher aggregates.

Significant differences in the intensity of fluorescence bands and small shifts in the emission maxima (λ_em_ in the range 565–573 nm) are observed in the fluorescence spectra of ligand **3** recorded in different solvents and aqueous solutions (Figure 2, Table 1). The fluorescence spectra for **3** in nonpolar 1,4-dioxane and toluene differ significantly from each other. In the first case, the fluorescence band exhibits a relatively high intensity comparable with that for aqueous solutions of **3**, while in toluene fluorescence is quenched. As ligand was strongly associated in both solvent (see UV-Vis spectra and Table 1) one can assume that these aggregates possessed different emission properties. It seems to be a characteristic feature for these carbazole-based compounds, because the same effects were observed for ligand carrying the triazole substituent [45]. Although both solvents are regarded as nonpolar, the different effect of 1,4-dioxane on the fluorescence of **3** may be explained by the structure of this solvent that can exhibit higher polarity in a boat-like conformation.

The 9-N-substituted carbazole derivatives were found to interact with G-quadruplexes via external stacking thanks to the presence of a planar carbazole unit and a vinylbenzothiazolium arm coupled with delocalized π-electrons [43,44,45]. The C=C double bond structural element of the ligand offered the opportunity of controlling the ligand structure by light (*cis-trans* photoisomerization), similarly as for the arylstilbazolium ligands [72]. The idea of synthesis of a photoisomerizable ligand was prompted by the search of ligands for possibile application in the dye-assisted DNA phototherapy.

It is known that the binding affinity of a ligand to DNA depends on the structure of the ligand (*trans*-*cis* isomerization) as well as on the topology of G-quadruplex. The structural factors play a crucial role in the molecular recognition of particular DNA forms, which can be employed for detection purposes, for example, some ligands recognize the parallel or antiparallel structures of G-quadruplexes. However, for the tested ligand **3** we were unable to confirm efficient photoisomerization. Despite the typical changes characteristic of transformation of isomer *trans* to *cis* detected in the absorption and fluorescence spectra, we did not observe the return to the initial state upon shorter wavelength irradiation of the isomeric mixture; the observed changes were irreversible, although the *trans* isomer is thermodynamically more stable. At the same time, the use of other techniques, such as NMR and HPLC, indicated that irradiation of ligand **3** solution with polychromatic light resulted in formation of small amounts of photoproducts due to other photochemical processes, such as photodegradation, photocyclization or photoaddition. Simultaneously, the stock solution of ligand (1.5 mM in DMSO) stored at 4 °C under light protection was stable for several months, which was controlled using UV-Vis and fluorescence techniques. For studies of ligand-DNA interactions, it is important to be sure, that the solution of the tested compound in the selected buffer is stable within the duration of the experiment. The solutions of ligand **3** (1 μM–50 μM) in Tris-HCl buffer were found to be sufficiently stable in time. No aggregation or precipitation phenomena were observed since the spectra recorded immediately and at 10 and 20 min after ligand addition perfectly overlapped and were indistinguishable. The ligand was also stable at higher temperatures as its spectra at different temperatures (20–95 °C) were recorded and revealed only slight differences in absorbance.

### 2.3. Spectrophotometric Titration

**Figure 3:**
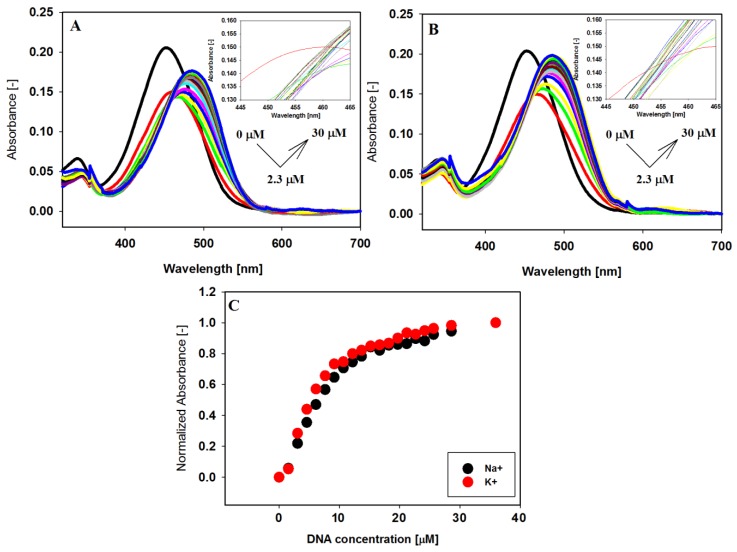
Spectrophotometric titration of ligand **3** (6 µM) with 22HT G4 (0–36 µM) in Tris-HCl buffer (10 mM, pH 7.2) containing 100 mM NaCl (**A**) and 100 mM KCl (**B**). Panel (**C**) shows normalized absorbance changes vs. increasing concentration of 22HT G-quadruplex at 499 nm (Na^+^) and 492 nm (K^+^).

Investigation of the interaction between the small carbazole ligand with human telomeric G-quadruplex DNA has started with the analysis of visible absorption spectra. The spectrophotometric titration experiments were conducted in a 10 mM Tris–HCl buffer (pH 7.2) containing 100 mM NaCl or KCl. The absorption spectrum of compound **3** changed significantly with increasing concentration of 22HT both in the antiparallel (NaCl) and hybride (KCl) form of the G-quadruplex (Figure 3).

The long wavelength maximum of the absorption spectrum of carbazole compound **3** at 453 nm was red shifted (Δλ = 33 nm), while the initially observed hypochromic effect (26–27%) changed the direction into a hyperchromic one (17–23%) (Table 2). The absence of sharp isosbestic points in the titration experiments indicated that what was being observed was more complex than simple two-component equilibrium between the free and bound ligand in the system (insets in Figure 3A,B).

Similar spectral effects observed in the UV-Vis titration experiments have been previously ascribed to a two-step complex formation between the ligand and G-quadruplex [43,44,73,74,75,76]. The hypochromic effect was observed at lower G4/ligand ratios, when the excess of positively charged ligand could be stacked onto the G-quadruplex surface as a result of electrostatic attraction with phosphate groups leading finally to ligand–ligand aggregation on the G-quadruplex. Under the influence of higher G4 concentrations the redistribution of ligand molecules occurred (aggregate dissociation) to the binding sites with higher affinity (the end-stacking mode). This process was manifested by a hyperchromic effect and red shift. The pronounced changes in the UV-Vis spectra indicate strong end-stacking interactions between the carbazole ligand and the external G-tetrads, which has been confirmed by CD studies (vide infra) [77,78]. The same effects were observed for the other carbazole ligands and the parallel G-quadruplexes c-MYC and c-KIT, which had the same type of interaction regardless of the G4 structure [43,44].

### 2.4. Fluorescence Spectroscopy

**Figure 4:**
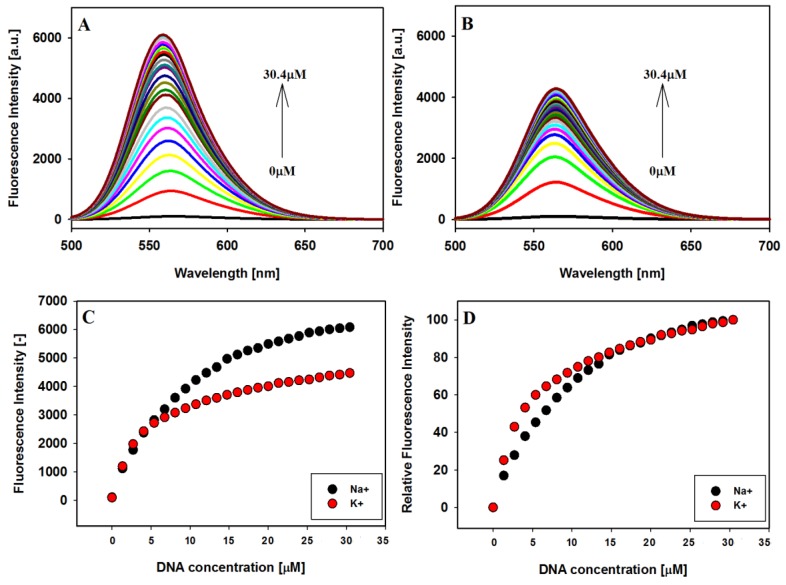
Fluorescence titration spectra of ligand **3** (2 µM) with G4 22HT in Tris-HCl buffer (10 mM, pH 7.2) containing 100 mM NaCl (**A**) and 150 mM KCl (**B**); fluorescence binding curves (**C**) and relative enhancement in the fluorescence intensities (**D**) of ligand vs. the increasing concentration of G-quadruplexes 22HT; λ_ex_: 486 nm in NaCl and 491 nm in KCl.

To further explore the interaction between carbazole ligand **3** and the studied G-quadruplexes, fluorescence spectroscopy was used. The emission spectra of the compound **3** were recorded in the presence of sodium and potassium ions with excitation at 486 and 491 nm, respectively. Fluorescence spectra recorded in the titration experiments and fluorescence titration curves are shown in Figure 4. Free ligand in Tris HCl buffer solution in the presence of NaCl or KCl, exhibited very low fluorescence as follows from the result of solvent effect study. With every addition of the 22HT G4 DNA emission of the ligand increased significantly, which may be related to the protection of the ligand from quenching by polar water molecules. The hydrophobic environment inside the G-quadruplex 22HT helps the ligand molecules towards the recovery of their fluorescence [37,79].

An alternative explanation for this phenomenon may be the deactivation of the excited state of compound in water through a low rotation energy barrier of benzothizolium moiety of ligand. The increase in fluorescence intensity of **3** upon end-stacking interaction with G4 results in the restriction of rotation around the single bond connecting the benzothiazolium and vinylcarbazole moieties of the dye [80]. The confirmation of this explanation may be the observation that the fluorescence intensity of free ligand also increases in the presence of 40% polyethylene glycol (PEG) solution, which possesses higher viscosity and causes the molecular crowding effect.

**Figure 5:**
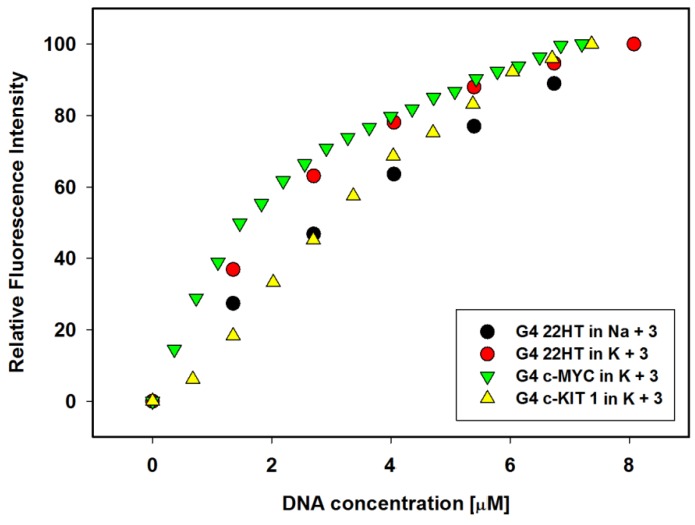
Comparison of the relative enhancement in the fluorescence intensities of ligand **3** vs. the increasing concentration of G-quadruplexes 22HT, c-MYC and c-KIT1.

The emission λ_max_ at 567 nm of free ligand in the presence of sodium cations underwent blue shift by 3 nm after the first addition of G-quadruplex and formation of the complex and by 8 nm after the last addition of DNA. At the same time, the maximum emission of free ligand in potassium buffer solution at 564 nm has not shifted (Figure 4A,B). The experiments were conducted until the changes in fluorescence spectra were insignificantly small. Interestingly, no plateau was observed for titration plots, similarly as we reported previously for other systems (Figure 5) [43,44]. The enhancement in the fluorescence intensity was more pronounced in the case of antiparallel G4 DNA formed in the presence of Na^+^ than for hybrid G4 in K^+^ ions, despite higher affinity of the ligand toward the G4 DNA stabilized with potassium ions. However, comparing the relative enhancement in the fluorescence intensities of ligand vs. increasing concentration of G-quadruplexes 22HT observed in the presence of sodium or potassium ions, one can notice a slightly higher effect in the presence of potassium cations (Figure 4D).

### 2.5. Binding Parameters of Ligand/22HT Complex

Using the UV-Vis and fluorescence titration experiments, the binding affinity of carbazole compound **3** to 22HT G4s was studied. Because saturation of binding curves in both methods was not achieved, the estimation of K values using the Scatchard equation was impossible. Instead, we decided to use the Benesi–Hildebrand method (B–H) for data analysis and estimation of the nK_b_ values (Table 3) [81]. It should be mentioned that in this method the stoichiometry of formed ligand/G4 complex is assumed 1:1, thus the result of the B–H calculation should be regard as a nK_b_ product.

Ligand **3** exhibited similar binding affinity for both G-quadruplexes, in sodium and potassium solution. The binding constants were calculated as 1.3–1.5 × 10^5^ M^−1^, using analysis of UV-Vis spectral data. The binding parameter calculated from the fluorescence titration experiment is in good agreement in the case of hybrid-mixed G-quadruplex obtained in the presence of potassium ions (Table 3). However, the binding product nK_b_ from fluorescence data for the antiparallel 22HT G-quadruplex in the presence of sodium ions was almost half lower than that obtained from absorbance measurements.

**Figure 6:**
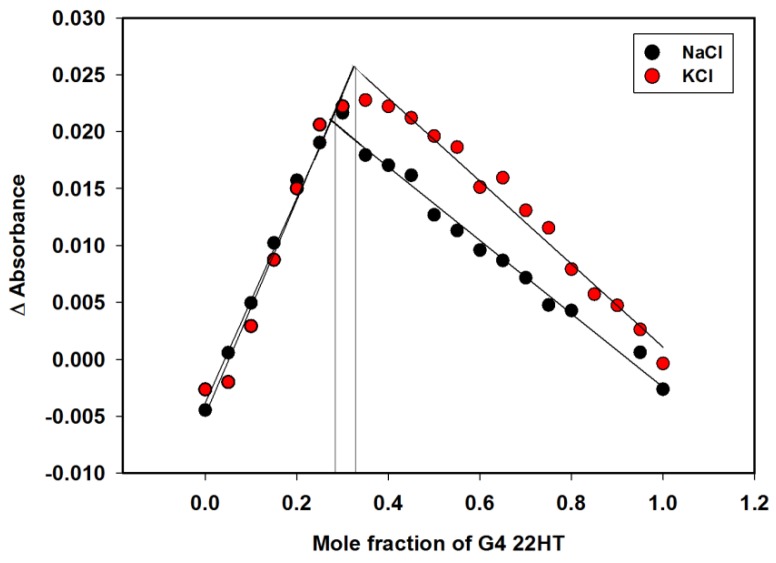
Job plots for the binding of compound **3** to 22HT G-quadruplex in Tris-HCl buffer containing 100 mM NaCl or KCl.

**Figure 7:**
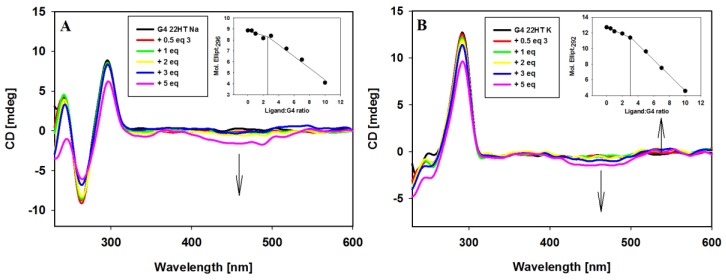
Circular dichroism (CD) spectra of G-quadruplex 22HT (5µM) with increasing amounts of ligand **3** in Tris-HCl buffer (10 mM, pH 7.2) containing 100 mM NaCl (**A**) and 100 mM KCl (**B**).

Binding stoichiometries with the G-quadruplex were then investigated applying an absorbance-based Job plot (Figure 6). In this method, the total molar concentration of carbazole and 22HT G4 were held constant, but their mole fractions were varied. Two sets of experiments were completed for each Job plot. As shown in Figure 6, inflection points in the graphs are observed at the DNA fraction of about 0.3 for complexes in sodium and potassium ions. This result indicated that ligand **3** could bind to 22HT G4 DNA by about 2:1 binding stoichiometry in the presence of sodium and potassium ions. These results were in agreement with CD analysis results (insets in Figure 7).

### 2.6. Circular Dichroism (CD) Spectroscopy

Circular dichroism (CD) spectroscopy is a powerful technique providing valuable information about the structure formation, stability, topology of G-quadruplexes, and on the effect of ligand interacting with G-quadruplex structures [82,83,84,85]. The typical CD spectrum of oligonucleotide with human telomeric sequence in the presence of Na^+^ ion shows a positive peak at 295 nm and two smaller peaks with the opposite orientation: a negative at 265 nm and a positive at 240 nm, characteristic of the antiparallel basket-type G-quadruplex structure [60]. In the presence of K^+^ ion G-quadruplex adopts a mixed-type hybrid structure with a characteristic CD spectrum showing a strong positive peak at 293 nm with a distinctive shoulder around 270 nm and a smaller negative peak at 240 nm [61,64].

The interactions of carbazole ligand **3** with telomeric G-quadruplexes in Na^+^ and K^+^ buffer solutions were studied. Upon the addition of ligand **3** to a sodium solution of G4 22HT, the intensity of the peaks changed, but their positions seemed to be stable. No significant change appeared at 295 nm even after 3 equiv. of ligand addition, only the intensity of the signal at 265 nm was slightly reduced. Addition of two more equivalents of compound **3** caused a 15% reduction of positive signal and did not cause significant changes in the intensity of the negative signal. The CD profile of G4 22HT recorded in K^+^ solution revealed that the addition of the ligand did not increase the shoulder at 270 nm, thus indicating that the preservation of G4 hybrid structure was independent of the excess of the ligand. Slight changes in the intensity of the bands at 293 and 255 nm were observed after the addition of 3 equiv. of ligand, while the addition of 2 additional equivalents caused a 28% reduction in intensity of the positive signal. Regardless of whether the experiments were carried out in the presence of Na^+^ or K^+^ ions, the induced negative signals (ICD) were observed in the long-wavelength region, in which the absorption bond of achiral carbazole ligand appears. This observation rather excludes the groove binding to G-quadruplex structures, the binding mode with characteristic positive ICD signals in the long-wavelength absorption region of the ligand (Figure 7A,B) [78,82,86,87]. Thus, the observed CD changes (ICD bands) indicate that the different G-quadruplexes such as c-MYC, c-KIT, KRAS and telomeric one bind to the studied compound in the same way irrespective of their structure and the results suggest a similar binding mode for this carbazole ligand [43,44]. It should be noted that in the spectra recorded in the presence of potassium ions, in addition to the negative induced signals (ICD), a weak positive induced band at 538 nm appeared (isoeliptic point ~500 nm) in the presence of smaller amounts of ligand (to 5 equiv.). Weak exciton splitting in the induced spectra indicates that the carbazole ligand can bind to the G-quadruplex grooves as one or maybe more stacked molecules [84]. This band disappeared in the presence of higher ligand concentration (7 and 10 equivalents), with the simultaneous deepening of the negative ICD intensity.

CD experiments were also carried out in order to prove binding stoichiometry of G4/ligand complexes. Spectral changes at the selected wavelengths (296 and 292 nm for G-quadruplexes in sodium and potassium ions) were plotted against ligand/DNA molar ratio (insets in Figure 7) to reveal about 2:1 stoichiometry of the complexes.

Furthermore, CD experiments were carried out to establish whether the G-quadruplex folding can be induced by ligand alone without addition of sodium or potassium ions. In the absence of salt, the CD spectrum of 22HT oligonucleotide at room temperature exhibited the negative bands at 238 and 278 nm, as well as a major positive band at 255 nm (random coil structure with a positive peak at 257 nm) [88].

**Figure 8:**
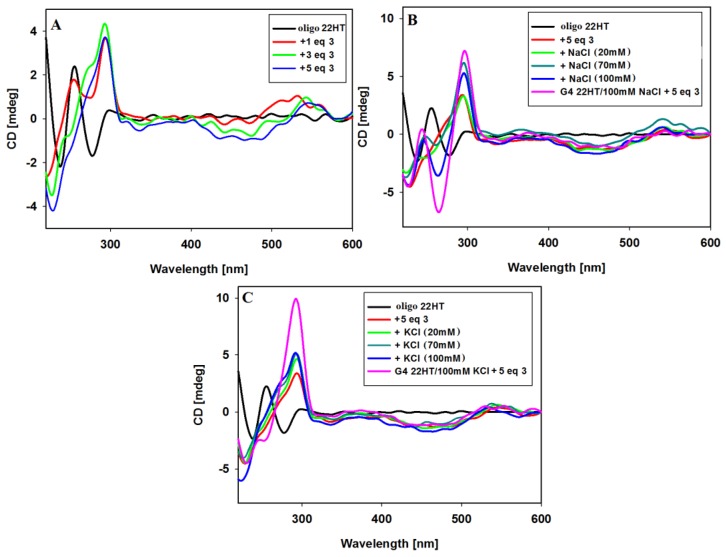
CD spectra of 22HT oligonucleotide (5µM) with increasing amounts of ligand **3** in Tris-HCl buffer (10 mM, pH 7.2) (**A**); CD spectra of 22HT oligonucleotide (5 µM) with 5 equiv. of ligand **3** in Tris-HCl buffer and increasing amounts (0–100 mM) of NaCl (**B**) and KCl (**C**).

A significant change in the CD spectrum was observed already after addition of 1 equivalent of ligand **3**. The immediate disappearance of the negative band at 278 nm was observed with the simultaneous formation of a positive intense band at 292 nm (Figure 8A). Further additives of up to 5 equiv. of ligand caused the disappearance of the positive band at 255 nm and the formation of a spectral pattern typical for mixed hybrid structure with a shoulder at about 270 nm. The spectrum remained unchanged within at least 24 h. This experiment was continued by the addition of sodium or potassium ions to such formed G4/ligand complex. NaCl and KCl were added in three portions to a final salt concentration of 20, 70 and 100 mM. As can be seen in Figure 8B, further NaCl additions caused structural changes and formation of an antiparallel G-quadruplex structure was observed. Upon addition of potassium ions, the shape of the spectra remained similar after the introduction of further portions of the salt, but the spectra differed slightly from those recorded initially for the structure of G4 prepared the day before, to which 5 equiv. of ligand was added. The differences is visible in the vicinity of 270 nm, which may indicate some conformational changes in the G-quadruplex DNA structure.

In these experiments (Figure 8), the induced signals (ICD) were also noticed in the long-wavelength region characteristic of achiral carbazole ligand **3.** After addition of 1 equiv. of ligand a positive induced signal was observed at 532 nm. Subsequent additions of ligand **3** (3 and 5 equiv.) caused an appearance of negative induced bands near 468 nm, and the induced positive bands were shifted toward longer wavelengths (at 550 nm, isoeliptic points at 524 nm) (Figure 8A). The addition of NaCl and KCl salts resulted finally in the same shape of ICD bands as those shown in Figure 7 (different order of reagent addition).

**Figure 9:**
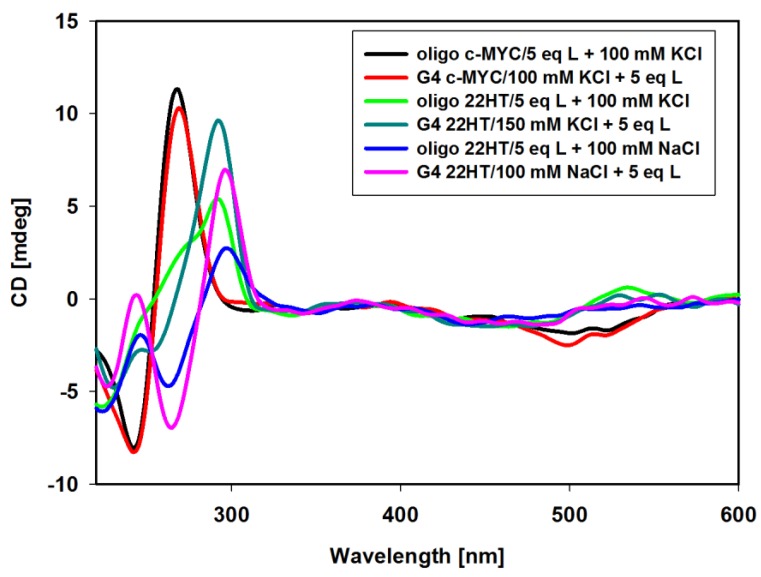
CD spectra of G-quadruplexes formed by 22HT and c-MYC oligonucleotides upon different addition order of ligand **3** and salt. Conditions: 5 µM 22HT or c-MYC, 25 µM ligand **3**, 100 mM KCl or NaCl, Tris-HCl buffer (10 mM, pH 7.2).

It should be also noted that the intensities of CD bands in G-quadruplex spectra formed in the presence of Na and K ions (Figure 7) are higher than those obtained for 22HT quadruplexes formed under the influence of ligand **3** and subsequent salt additions (Figure 8). It should also be emphasized that such an effect was not observed in the CD spectra of G-quadruplex formed by the DNA sequence derived from the NHE III_1_ region of c-MYC. In this case, the same CD spectra were observed irrespectively of the reagent addition order (potassium ions and ligand **3**) and the spectra overlapped very well (Figure 9).

**Figure 10:**
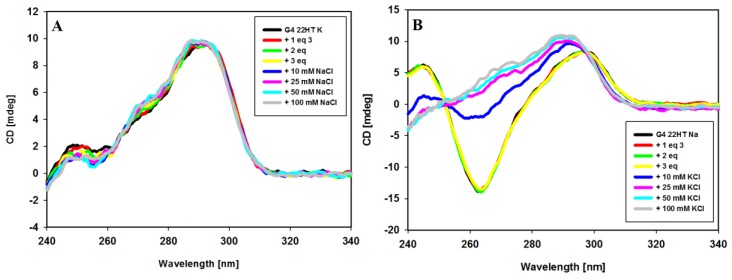
CD spectra of G-quadruplex 22HT (3µM) in the cation replacement experiments. Conditions: Tris-HCl buffer (10 mM, pH 7.2) containing 100 mM NaCl (**A**) and 100 mM KCl (**B**) with increasing amounts of ligand **3** and subsequent increasing amounts of competing cation.

In the next experiment, we intended to verify whether metal cations (Na^+^, K^+^) can be replaced by each other in the already formed G4/ligand complex. Results are shown in Figure 10. The CD spectrum of the potassium-22HT hybrid G-quadruplex formed in the presence of potassium and 3 equiv. of ligand **3** did not change upon addition of Na^+^ cations (up to 100 mM) as shown in Figure 10A, while the addition of K^+^ cations to the antiparallel G-quadruplex formed in the presence of sodium cations and ligand **3** caused significant spectral changes in CD bands. Observed spectral changes in Figure 10B suggest a structural rearrangement leading to a final hybrid structure of G4. This indicated that sodium cations did not easily displace the potassium ions from their positions in the G-quadruplex channel. Similar processes were reported for G-quadruplexes in the absence of ligands and this means that ligand **3** has no influence on this process [61].

### 2.7. DNA Melting Studies

**Figure 11:**
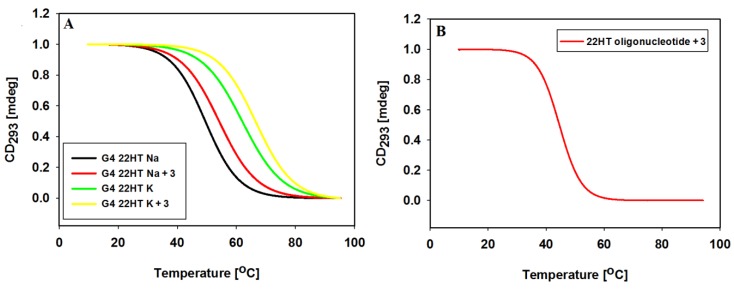
Normalized CD melting profiles of 22HT G-quadruplex at 293 nm without and with 3 equiv. of ligand **3** in 10 mM Tris-HCl buffer (pH 7.2) containing 100 mM NaCl and 100 mM KCl (**A**); Panel (**B)** shows normalized CD melting profiles of 22HT oligonucleotide at 293 nm with 3 equiv. of ligand **3** in 10 mM Tris-HCl buffer (pH 7.2).

To determine thermal stabilization of G4s 22HT DNA formed in the presence of ligand **3** and 100 mM NaCl or KCl, the temperature-dependent changes in the CD spectra were monitored in 10 mM Tris-HCl buffer (pH 7.2). Melting was plotted for the wavelength of 293 nm, at which there is the characteristic positive CD signal assigned to the typical antiparallel and hybrid G-quadruplex telomeric structures (Figure 11, Table 4).

The melting curves of 22HT G-quadruplex in the presence of Na^+^ ions, exhibited a small hysteresis in the reverse scan, while hysteresis almost disappeared after addition of ligand **3**. In the presence of potassium ions melting and annealing processes proceeded without hysteresis indicating the same kinetics both without and with ligand **3**. We also studied thermal stability of the G-quadruplex/ligand complex through measurements of the melting profile of the 22HT oligonucleotide incubated with carbazole ligand in the absence of Na^+^ and K^+^ ions.

The positive ΔT_m_ values obtained confirmed previously reported results that tested ligand shows similar stabilizing effect toward the K^+^ and Na^+^-based G4 DNA (Table 4). Furthermore, the stability of G-quadruplex/ligand **3** complex formed in the absence of metal cations clearly indicates that ligand **3** can induce folding of 22HT into the G-quadruplex structure (Figure 11B, Table 5).

### 2.8. Biological Activity

**Figure 12:**
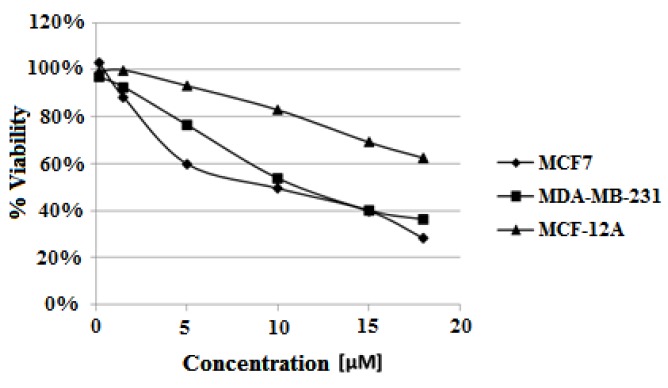
Dose–effect curves for studied cell lines treated with different concentrations of compound **3** (concentration range of 0.15–18 μM) for 72 h.

Cytotoxic study was carried out according the procedure described in Experimental using the highest ligand concentration of 18 μM. The derivative of carbazole **3** decreased both estrogen-dependent MCF7 and estrogen-independent MDA-MB-231 cells viability. The ligand showed higher cytotoxic activity against MCF7 cells than for MDA-MB-231 after 48 h (IC_50_ = 12.5 μM and 13.4 μM, respectively) and 72 h (IC_50_ = 9.5 μM and 11.4 μM). Interestingly the slightest viability reduction was observed for non-tumorigenic MCF-12A cells. Viability of MCF-12A cells after 72 h treatment with 18 μM ligand was 62%, compared to 28% for MCF7 and 36% for MDA-MB-231 cells (Table 5, Figure 12).

**Figure 13:**
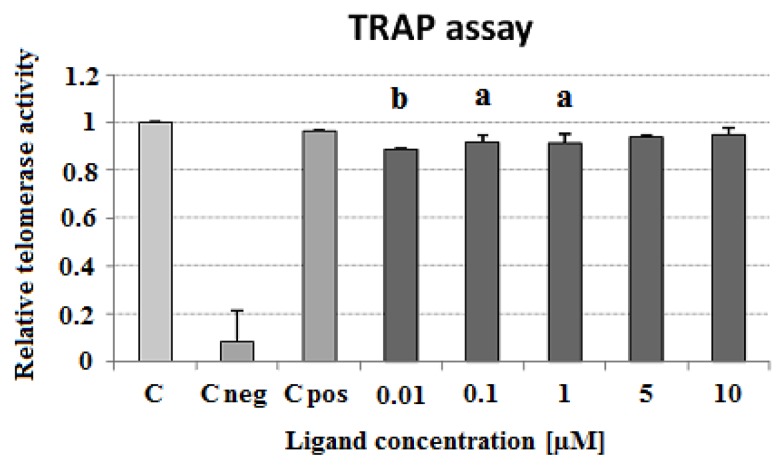
Influence of carbazole derivative **3** on telomerase activity in a cell-free telomerase repeat amplification protocol (TRAP) assay. C, control cell extract; C neg, negative cell extract; C pos, positive cell extract. *p* < 0.05 (a), *p* < 0.01 (b).

**Figure 14:**
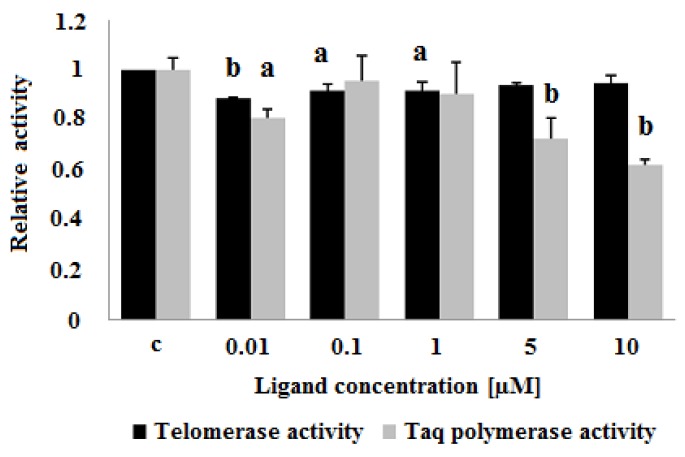
Influence of carbazole derivative **3** on telomerase activity in a cell-free TRAP assay and on Taq polymerase activity. C, control cell extract. *p* < 0.05 (a), *p* < 0.01 (b).

The ligand-induced inhibition of telomerase activity was assessed by a telomerase repeat amplification protocol assay (TRAP) as described in Experimental. In the ligand concentration range of 0.01–10 μM the carbazole derivative **3** slightly decreased the activity of telomerase in cell-free assay. Surprisingly, lower concentrations (0.01 and 0.1 μM) showed a greater ability for telomerase inhibition (89 and 92% compared to control cell extract, respectively) (Figure 13). To assess the influence of ligand **3** on Taq polymerase activity and to verify the specific effect on telomerase activity, a polymerase chain reaction (PCR) experiment in the presence of different compound concentrations was performed. The PCR revealed a decrease in Taq polymerase activity at the higher concentrations of the ligand (5 and 10 μM) (Figure 14).

In conclusion, the derivative of carbazole **3** shows significantly higher cytotoxicity against breast cancer cells then non-tumorigenic breast epithelial cells. The cytotoxic activity of ligand seems to be not associated with telomerase inhibition. However, many known G-quadruplex stabilizing ligands do not target the telomerase enzyme, but the telomere itself [8,90,91,92].

## 3. Conclusions

Carbazole derivative **3** was investigated for its interaction with human telomeric (22HT) G-quadruplex DNA in Na^+^ and K^+^-containing buffer using several methods. Our results indicated that tested compound **3** could stabilize the antiparallel and hybrid G-quadruplex structures and could induce the hybrid G-quadruplex structure formation in the absence of metal cations. All methods showed that investigated ligand apparently has higher affinity to telomeric DNA in the presence of potassium ions. The spectrophotometric titration results have shown a two-step complex formation between ligand and G-quadruplex that was manifested by consecutive the hypochromic and hyperchromic effects and red shift. The pronounced changes in the UV-Vis spectra indicate strong end-stacking interactions between the carbazole ligand and the external G-tetrads, which has been confirmed by the induced CD band of ligand. Large spectral changes in ligand spectra at higher G4 concentrations have been observed also in case of fluorescence titration experiments. The ligand **3** exhibiting poor fluorescent properties in an aqueous solution, showed a strong fluorescence enhancement upon binding to G-quadruplex. Two explanations of this phenomenon may be taken into account: the effect of the hydrophobic environment inside the G-quadruplex 22HT and inhibition of rotation of benzothiazolium moiety during end-stacking interaction with G-quadruplex. The derivative of carbazole **3** shows significantly higher cytotoxicity against breast cancer cells then for non-tumorigenic breast epithelial cells. The cytotoxic activity of the ligand seems to be not directly associated with telomerase inhibition as indicated from TRAP assay results.

## 4. Experimental

### 4.1. Materials

#### 4.1.1. Ligands

##### General Procedure for Synthesis of Carbazole Derivatives

*9-(4-(1H-imidazol-1-yl)butyl)-9H-carbazole-3-carbaldehyde***2.** To *N,N*-dimethylformamide (1.67 mL, 21.28 mmol, 24.3 equiv) cooled at 0 °C, phosphoryl chloride (1.69 mL, 18.44 mmol, 20.7 equiv.) was added dropwise. The mixture was stirred for 30 min and then heated to room temperature and stirred for 1.5 h. The carbazole derivative **1** (258 mg, 0.89 mmol) in 1,2-dichloroethane (1 mL) was added. The mixture was heated at 90 °C for 32 h. After cooling to rt, mixture was poured into ice and after room temperature was reached, the phases were separated and the aqueous one was extracted with chloroform/2-propanole (3:1) three times (40 mL each). The aqueous solution was neutralized by 25% NaOH and then extracted with chloroform/2-propanole (3:1) three times (40 mL each). This organic phase was washed with water and combined with the first organic solution. After the standard work-up of organic phase, the crude product **2** was purified by column chromatography (crude product: silica gel, 1:20 methanol:dichloromethane = 1.2%) to give monoaldehyde derivative **2** in 29%. Mp 102–105 °C; IR $\tilde{\nu }$/cm^−1^: 2862, 2817 (C-H), 1686 (C=O); ^1^H-NMR (500 MHz) δ ppm: 1.73 (s, 4H, -CH_2_C**H_2_**C**H_2_**CH_2_-), 3.96 (dd, *J* = 6.4, 5.9 Hz, 2H, -C**H_2_**-imidazole), 4.48 (dd, *J* = 6.4, 5.9 Hz, 2H, -C**H_2_**-carbazole), 6.84 (s, 1H, imidazole 4-**H**), 7.09 (s, 1H, imidazole 5-**H**), 7.29–7.32 (m, 1H, Ar-**H**), 7.53 (t, *J* = 7.8 Hz, 1H, Ar-**H**), 7.56 (s, 1H, imidazole 2-**H**), 7.70 (d, *J* = 8.2 Hz, 1H, Ar-**H**), 7.79 (d, *J* = 8.5 Hz, 1H, Ar-**H**), 7.99 (d, *J* = 8.5 Hz, 1H, Ar-**H**), 8.30 (d, *J* = 7.8 Hz, 1H, Ar-**H**), 8.76 (s, 1H, Ar-**H**), 10.06 (s, 1H, C**H**O); EI-MS *m*/*z* (%): 318 (M + 1, 10), 317 (M^+^, 41), 289 (19), 208 (81), 180 (53), 109 (14), 58 (10); high-resolution mass spectrometry (HRMS) found: 317.15424; calcd for C_20_H_19_N_3_O: 317.15280.

*(E)-2-(2-(9-(4-(1H-imidazol-1-yl)butyl)-9H-carbazol-3-yl)vinyl)-3-ethylbenzo[d]thiazol-3-ium***3**. To a stirred solution of 9-(4-(1*H*-imidazol-1-yl)butyl)-9*H*-carbazole-3-carbaldehyde **2** (63.5 mg, 0.2 mmol) in MeOH (10 mL) solution of 3-ethyl-2-methyl-benzothiazolium iodide (61 mg, 0.2 mmol, 1.0 equiv.) in MeOH (10 mL) was added. The mixture was stirred at ambient temperature until completion of the reaction (TLC, 4 h). The solid was filtered off, washed with hexane and dried to give 65.5 mg (Y 54%) of an HPLC-pure intensely orange *trans*-isomer **3** used without further purification. Mp 232–235 °C; IR $\tilde{\nu }$/cm^−1^: 1581 (C=N), 957 (*E* CH=CH); ^1^H-NMR (DMSO-*d*_6_, 700 MHz) δ ppm: 1.51 (t, *J* = 7.2 Hz, 3H, N-CH_2_-C**H_3_**), 1.73-1.77 (m, 2H, -CH_2_CH_2_C**H_2_**CH_2_-), 1.80–1.84 (m, 2H, -CH_2_C**H_2_**CH_2_CH_2_-), 4.08 (t, *J* = 6.7 Hz, 2H, -C**H_2_**-imidazole), 4.52 (dd, *J* = 6.7, 7.0 Hz, 2H, -C**H_2_**-carbazole), 4.98 (q, *J* = 7.2 Hz, 2H, N-C**H_2_**-CH_3_), 7.20 (s, 1H, imidazole 4-**H**), 7.34–7.37 (m, 2H: 1H Ar-**H**, 1H imidazole 5-**H**), 7.56 (t, *J* = 7.7 Hz, 1H, Ar-**H**), 7. 72 (d, *J* = 8.3 Hz, 1H, Ar-**H**), 7.78 (dd, *J* = 7.4, 8.0 Hz, 1H, Ar-**H**), 7.80 (d, *J* = 8.7 Hz, 1H, Ar-**H**), 7.87 (dd, *J* = 7.7, 8.0 Hz, 1H, Ar-**H**), 8.02 (d, *J* = 15.4 Hz, 1H, C**H**=CH), 8.22–8.24 (m, 3H:2H Ar-**H**, 1H imidazole 2-**H**), 8.27 (d, *J* = 8.3 Hz, 1H, Ar-**H**), 8.42 (d, *J* = 15.4 Hz, 1H, CH=C**H**), 8.43 (d, *J* = 7.7 Hz, 1H, Ar-**H**), 8.93 (s, 1H, Ar-**H**); TOF-MS *m*/*z* (CH_3_OH): [M]^−^ 127 *m*/*z*, [M]^+^ 477 *m*/*z*, multiply-charged ions: [M + 1]^2+^ 239 *m/z*. HRMS found: 477.2014; calcd for C_21_H_19_N_3_O_2_: 477.2113.

#### 4.1.2. Oligonucleotide

The quadruplex-forming 22-mer deoxyribonucleotide with human telomeric sequence 5′-AGGG(TTAGGG)_3_-3′ (22HT) was purchased from Genomed (Warszawa, Poland) and was used without further purification. The strand concentration was determined at 260 nm at 85 °C using extinction coefficient of 251,800 M^−1^·cm^−1^ as calculated from the published values of molar absorptivities of nucleotides [93]. Before using, the oligonucleotide solution was heated at 90 °C for 5 min and subsequently allowed to slow cooling to room temperature, and stored at 4 °C overnight.

### 4.2. Methods

#### 4.2.1. Absorption Spectroscopy

The absorption spectra were recorded on a Cecil CE-2021 spectrophotometer (Cambridge, UK) in the 200–700 nm range at 25 °C. All measurements were carried out using a 10 mm quartz cell. UV–Vis absorption titrations were carried out by the stepwise addition of 1 µL aliquots of 1530 µM/strand of G4 DNA 22HT solution to a cell containing 1000 µL of 6 µM ligand. Three minutes was the equilibration time after each DNA addition. All measurements were performed in a 10 mM Tris–HCl buffer (pH 7.2) containing 100 mM NaCl or KCl.

#### 4.2.2. Continuous Variation Analysis

Job plot was used to obtain stoichiometry of the ligand **3**/22HT G4 complex. Two experiments were performed for each Job plot. In the first one 1 mL of 5µM carbazole solution was placed in two absorption cuvettes, one of which was used as reference. 50 µl additions of 22HT G4 solution were added to the sample cuvette, and the same volumes of buffer were added to the reference cuvette. In the second experiment, 1 mL of 5 µM 22HT G4 was placed in the sample cell and 1 mL of 10 mM TrisHCl buffer was placed in a reference cell. Both cuvettes were titrated with ligand solution (5 µM). Each sample was stirred vigorously and incubated for 3 min to allow equilibration. The difference absorption spectra were collected in 300–700 nm range at 25 °C with a 1 cm path-length quartz cell. Job plots were constructed by plotting the absorbance at 505 nm versus mole fraction of G-quadruplex.

#### 4.2.3. Fluorescence Spectroscopy

The fluorescence measurements were carried out using a Jasco FP 8200 spectrofluorimeter (Tokyo, Japan). The sample solution was thermostated at 25 °C. All measurements were carried out using a 10 mm quartz cell. The fluorescence spectra were collected from 500 to 700 nm with both excitation and emission slits being 5 nm. Fluorescence titrations were carried out by the stepwise addition of 1 µL aliquots of 1350 µM/strand of 22HT solution to a cell containing 1000 µL of 2 µM ligand in buffer. Three minutes was the equilibration time after each DNA addition followed by emission spectrum recording. All measurements were performed in a 10 mM Tris–HCl buffer (pH 7.2) containing 100 mM NaCl or KCl. Excitation wavelength (λ_ex_) was set at 486 nm for NaCl and at 491 nm for KCl solutions.

#### 4.2.4. Circular Dichroism

CD spectra were measured on a Jasco J-810 spectropolarimeter (Jasco, Tokyo, Japan), in the spectral range from 210 to 600 nm with a 500 nm/min scan speed and a bandwidth of 1 nm. Spectra were recorded in quartz cuvettes of 1 cm path length and were averaged from 3 scans. Measurements with oligonucleotide were performed at 25 °C in a 10 mM Tris–HCl buffer (pH 7.2) containing 100 mM NaCl or KCl. Concentration of 22-mer oligonucleotide 22HT was 5 µM/strand. Ligand **3** was added to G4 DNA solution at increasing concentration from 0.1 to 5 molar equivalents.

In the melting studies, the temperature of the samples was maintained by a Jasco Peltier temperature controlled cell holder. Samples of the 22HT G-quadruplex for melting profiles were prepared by heating the 2 μM oligonucleotide solution in 10 mM Tris HCl buffer (pH 7.2) and 100 mM NaCl or 100 mM KCl at 90 °C for 5 min followed by slow cooling, and storing at 4 °C overnight. The melting profiles were recorded in the absence and presence of 3 equiv. of ligand **3** in 10–95 °C range with a 0.5 °C/min temperature gradient. All experiments were carried out using quartz cuvettes with a 10 mm optical path. Data were collected at 293 nm using both heating and cooling approaches. Typically three replicate experiments were performed, and average values of melting temperature are reported.

#### 4.2.5. Ligand 3—22HT G4 Binding Study

Binding data obtained from spectrophotometric and spectrofluorimetric titrations were analyzed according to Benesi-Hildebrand transformation [81]. Experiments were carried out in the same manner—after each G4 DNA addition, the titrated solution was incubated for 3 min followed by the UV-Vis or fluorescence spectrum measurement. The titration was continued until only small changes in the absorption or fluorescence spectra were observed upon successive addition of 22HT G4.

The method of Benesi–Hildebrand, used to estimate the value of nK_b_, is based on the Equations (1) and (2) that describe the one-site ligand binding model:

(1) $$\frac{c}{A-A_{0}}=\frac{c}{A_{m}-A_{0}}+\frac{c}{(A_{m}-A_{0}){\mathrm{nK}}_{b}}\cdot \frac{c}{{\mathrm{cG}}_{4}\cdot \mathrm{DNA}}$$

(2) $$\frac{1}{F-F_{0}}=\frac{c}{F_{m}-F_{0}}+\frac{c}{(F_{m}-F_{0}){\mathrm{nK}}_{b}}\cdot \frac{c}{{\mathrm{cG}}_{4}\cdot \mathrm{DNA}}$$

where c is the concentration of ligand, A_0_ is the absorbance of ligand in the absence of G4 DNA, A is the absorbance recorded in the presence of added G4 DNA, Am is absorbance at saturation in presence of added [G4 DNA]max, Fo is the fluorescence of ligand in the absence of G4 DNA, F is the fluorescence recorded in the presence of added G4 DNA, Fm is fluorescence at saturation in presence of added [G4 DNA]max, and n is the number of bound ligand molecules per G-quadruplex, K_b_ is the binding constant. The value of nK_b_, was obtained from a ratio of intercept and slope of plot (Equation (1) or (2)).

#### 4.2.6. Biological Activity

##### Cytotoxic effect–MTT assay

An estrogen-dependent human MCF-7 and estrogen-independent human MDA-MB-231 breast cancer cells, were cultured in RPMI1640 medium (Biochrom GmbH, Merck Millipore, Germany) supplemented with 10% fetal bovine serum. A non-tumorigenic epithelial human mammary MCF-12A cells were cultured in DMEM/F-12 medium. Before reaching confluence, cells were counted and passaged into 96-well plates (5000 cells per well). Cells were cultured for 24, 48 or 72 h with or without ligand at 0.15–18 μM. The solvent, DMSO in a concentration of 0.10%, was also applied as a control. After this time 10 μL of MTT solution (5 mg/mL) (Sigma–Aldrich, St. Louis, MO, USA) was added to each well. The plate was incubated at 37 °C for 4 h followed by 100 μL of solubilization buffer (10% SDS in 0.01 M HCl) addition. Cell viability was quantified spectrophotometrically using a Labsystems Multiscan RC plate reader (Thermo Fisher Scientific, Helsinki, Finland). Cytotoxicity rate was expressed as the absorbance of a sample comparing to control cells, IC_50_ values were calculated using CalcuSyn (Biosoft, Cambridge, UK) and the standard deviation was calculated using Excel software (Microsoft, Redmond, WA, USA). Each experiment was repeated at least three times. All experiments were performed in minimal exposure to light.

##### Telomerase Repeat Amplification Protocol (TRAP) Assay

The inhibitory activity of ligand **3** against human telomerase in a cell-free assay was assessed using the quantitative TeloTAGGG Telomerase PCR ELISA kit (Roche Diagnostics, Indianapolis, IN, USA), a modified original TRAP. Briefly, a cell extract was prepared from exponentially growing MCF-7 cells according to manufacturer’s protocol. For each assay, cell extract corresponding to 1 µg of total protein was used. The reaction mixtures contained telomerase extract, ligand **3** in different concentrations 0.01–10 µM, biotin-labeled synthetic P1-TSand P2 primers for amplification of telomeric repeats, Taq DNA polymerase (5 units/μL) (Roche Diagnostics, Indianapolis, IN, USA), dNTP Mix and TRAP reaction buffer. After 20 min incubation at 25 °C for telomerase extension of the P1-TS primer, the PCR cycling conditions were 94 °C for 5 min followed by 30 cycles at 94 °C for 30 s, 50 °C for 30 s and 72 °C for 90 s with final step at 72 °C for 10 min. The PCR products derived from telomerase elongation were quantitated using an enzyme-linked immunosorbent assay (ELISA) and measurement of the absorbance at A450 nm against blank reference at A690 nm (Labsystems Multiscan RC; Helsinki, Finland). A heat-inactivated cell extract was also tested as a negative control.

## Figures and Tables

**Table 1:** Effect of solvent on the spectral parameters in the absorption and fluorescence spectra of ligand **3** (mean values ± standard deviation (SD) of three determinations).

Solvent	ε/10^4^ [M^−1^∙cm^−1^]	Abs. λ_max_ [nm]	Em. λ_max_ [nm]
CH_2_Cl_2_	5.2 ± 0.1	501	566
EtOH	5.0 ± 0.1	469	569
CHCl_3_	5.1 ± 0.1	504	566
MeOH	4.9 ± 0.1	479	569
ACN	4.8 ± 0.1	473	570
H_2_O	4.3 ± 0.1	451	565
10 mM Tris-HCl	4.3 ± 0.1	451	566
DMSO	4.3 ± 0.1	468	573
1,4-Dioxane	1.9 ± 0.1	482	566
Toluene	1.9 ± 0.1	452	565

**Table 2:** Spectral effects for ligand **3** bound to 22HT G-quadruplexes.

DNA	Δλ_max_ [nm] ^a^	Hypochromicity [%] ^b^	Hyperchromicity [%] ^b^
G4 22HT Na^+^	33	27	17
G4 22HT K^+^	33	26	23

^a^ Δλ batochromic shift. ^b^ Hypochromicity and hyperchromicity were measured at λ_max_. The hypo- and hyperchromicity percentages were calculated using following equations: %Hypo = [(A_f_ − A_b, min_)/A_f_] × 100 and %Hyper = [(A_b, max_ − A_b, min_)/A_b, max_] × 100, where A_f_, A_b, min,_ A_b, max_ are the absorbance values of free ligand and of bound ligand at minimum and maximum (excess of G4), respectively.

**Table 3:** Parameters for the interaction of ligand **3** with G-quadruplex 22HT determined using the Benesi–Hildebrand method from absorption and fluorescence titration data (K_b_—binding constant, n—number of bound ligand molecules per G-quadruplex).

Cations	Benesi–Hildebrand Method, nK_b_ (× 10^5^ M^−1^)	
	Spectrophotometric Titration	Fluorescence Titration
Na^+^	1.3 ± 0.1	0.8 ± 0.1
K^+^	1.5 ± 0.3	1.7 ± 0.3

**Table 4:** Results of the DNA melting studies.

Cations	T_m_ [°C]	T_m_ [°C]	ΔT_m_ [°C] ^e^
Na^+^	49.5 ^a^	54.1 ^c^	4.5
K^+^	62.0 ^a^	66.3 ^c^	4.3
----	<14 ^b^	44.6 ^d^	>30.6

^a^ T_m_ of 22HT G4 in the presence of 100 mM NaCl and KCl in 10 mM Tris-HCl buffer (pH 7.2) (lit. 56 °C in 100 mM NaCl and lit. 63.0 °C in 100 mM KCl in a pH 7.0, 10 mM sodium cacodylate buffer [89]). ^b^ T_m_ of oligonucleotide 22HT in the absence of salt in 10 mM Tris-HCl buffer (pH 7.2). ^c^ T_m_ of 22HT G4 incubated with 3 equiv. of ligand **3**. ^d^ T_m_ of oligonucleotide 22HT incubated with 3 equiv. of ligand **3** in the absence of salt. ^e^ ΔT_m_ was obtained from the differences in the melting temperatures of the 3 equiv. of ligand bound and uncomplexed with DNA. Data were collected at 293 nm. Typically three replicate experiments were performed, and average values are reported with a standard deviation of ±0.5 °C.

**Table 5:** In vitro cytotoxicity of carbazole derivative **3** on selected cell lines.

Cytotoxicity (IC50, [μM])			
Cell Line	Time Interval		
	24 h	48 h	72 h
MCF7	>18	12.5	9.5
MDA-MB-231	>18	13.4	11.4
MCF-12A	>18	>18	>18
